# Genomic Connectivity and Adaptive Gene Flow of *Anopheles gambiae s. s*. Across East and Central Africa

**DOI:** 10.21203/rs.3.rs-10571451/v1

**Published:** 2026-10-01

**Authors:** Fekadu Gemechu, Kelly L. Bennett, Araya Eukubay, Sisay Dugassa, Anastasia Hernandez-Koutoucheva, Luna Kamau, Tadesse Kebede Zeleke, Alistair Miles, Deriba Abera, Chris S. Clarkson, Lemu Golassa

**Affiliations:** 1 Aklilu Lemma Institute of Health Research, Center for Pathobiology, Addis Ababa University, Addis Ababa, Ethiopia; 2 Ethiopian Public Health Institute, Addis Ababa, Ethiopia; 3 Liverpool School of Tropical Medicine, Pembroke Place, Liverpool, L3 5QA, UK; 4 Ellison Institute of Technology, Littlemore, Oxford, OX4 4GE, UK; 5 College of Natural and Computational Sciences, Department of Zoological Sciences, Addis Ababa University, Addis Ababa, Ethiopia; 6 Department of Microbiology, Immunology, and Parasitology, College of Health Science, Addis Ababa University, Addis Ababa, Ethiopia.; 7 Centre for Biotechnology Research and Development, Kenya Medical Research Institute, Kenya

**Keywords:** *Anopheles gambiae s.s.*, genomic insecticide resistance, Adaptive gene flow, geographical population connectivity, whole-genome sequencing, malaria, Ethiopia, East-Central Africa

## Abstract

Population connectivity influences the adaptive capacity of disease vectors by mediating the spread of insecticide resistance alleles. High gene flow poses challenges for tailored intervention strategies targeting localised populations, but connectivity is often poorly characterised for particular geographical regions. This study uses whole-genome data to investigate the population connectivity of the *Anopheles gambiae* malaria vector across East-Central Africa, including understudied populations from Ethiopia. We also investigated adaptive gene flow using Diplotype clustering at known insecticide resistance loci. Population structure analyses revealed that Ethiopian populations experience high gene flow with inland East African *An. gambiae* including Uganda, inland Kenya, inland Tanzania, and the northern DRC, likely reflecting ecological continuity across the region. In contrast, coastal populations of East Africa and south-central DRC were subject to restricted gene flow, consistent with geographical and ecological barriers to dispersal. Diplotype clustering of the *Cyp6aa/p* gene cluster and *Cyp9k1* revealed that CNV-variant sharing broadly mirrored genome-wide connectivity patterns, with shared variation present in inland East Africa and the northern DRC, but distinct CNV-associated clusters in more isolated populations in southeastern DRC. On the other hand, allele sharing at the *Vgsc* gene did not follow patterns of population connectivity, with the *Vgsc*-L995S allele broadly shared across East Africa and the *Vgsc*-L995F allele shared between Ethiopia and populations throughout the DRC. Notably, Ethiopia emerges as a crossover point where resistance alleles from East and Central Africa can converge, suggesting it is an important region for the monitoring of emergent and/or expanding adaptations. Findings highlight the importance of cross-border collaboration and the development of resistance management strategies that respond to both regional connectivity and local divergence.

## Introduction

Population connectivity impacts the capacity of disease vectors to adapt and persist under changing ecological conditions, and under selection pressure from population control interventions^1,2^. This is because connectivity is a factor in the speed at which adaptive traits spread across geographical regions^3,4^ For example, in malaria vector mosquitoes, gene flow within and between species facilitates the spread of insecticide resistance alleles, which can disseminate widely, compromising vector control strategies^5,6^. While high levels of connectivity can accelerate this process, ecological and geographical barriers to vector dispersal may slow or redirect it, creating heterogeneous patterns of resistance distribution^1^. Within the major malaria vector *Anopheles gambiae s.s*. populations, connectivity among geographically separated groups complicates efforts to contain resistance to insecticide-treated bed nets and indoor residual sprays^7–9^, which remain the most widespread preventive tools against malaria transmission^10^. Examining patterns of gene flow within this species across East Africa is therefore essential to identify the dispersal corridors of adaptive alleles^1,7,11^ and design surveillance and/or control programmes that anticipate the spread of resistance across the heterogeneous environments in this region.

*Anopheles gambiae s.s*. (hereafter *An. gambiae*) is recognised as the primary vector of malaria in many East African countries, alongside the other major species, *An. funestus* and *An. arabiensis*^12,13^. In addition, *An. coluzzii* is important in East and Central African countries, including the Democratic Republic of the Congo, Kenya and Sudan, where its distribution and resistance dynamics have been documented^14–16^. The country of Ethiopia has long been considered unique in that *An. arabiensis* was regarded as the dominant vector^17^ with *An*. *funestus, An. nili,* and *An. pharoensis* serving as secondary vectors^18,19^, but with no comprehensive reports of *An. gambiae* populations, which are otherwise widespread across East and Central Africa^20–22^. However, recent genomic analyses have confirmed its presence in western Ethiopia^23^ after a small number of *An. gambiae* were first detected by a molecular assay in central Ethiopia^24^. Its highly efficient vectorial capacity is driven by a combination of ecological and behavioural traits, including strong anthropophily^25,26^ and a tendency for indoor biting and resting; high reproductive potential; and ecological plasticity^27–29^. These attributes are important because they enable *An. gambiae* to persistently exploit human environments and sustain transmission even under intensive intervention pressure^30^.

Because this taxon has only recently been described from Ethiopia, nothing is yet known about its population connectivity, including whether it experiences gene flow with populations in neighbouring countries. However, studies from East and Central Africa show that inland *An. gambiae* populations often exhibit extensive gene flow and low differentiation across wide geographical areas. In contrast, coastal and central African populations may display a greater divergence due to ecological speciation in coastal habitats, geographical barriers such as river basins and mountain terrains, and demographic bottlenecks^7,31,32^. Furthermore, evidence from whole-genome studies suggests this has impacted the sharing of insecticide resistance alleles^5^. Insecticide resistance in *An. gambiae* is now widely distributed across Africa^33–35^.

The major resistance mechanisms impacting the genome include target-site substitutions in the voltage-gated sodium channel gene (*kdr* alleles)^36,37^ that confer resistance to pyrethroids and DDT and metabolic detoxification mediated by the amplification of cytochrome P450 monooxygenases (*Cyp*), glutathione S-transferases (*Gste*), and carboxylesterase (*Coeae*) genes^38–41^. Recent genomic surveillance evidence has shown the geographical spread and rising frequency of known and novel single-nucleotide polymorphisms (SNPs), such as *Vgsc-*L995F*/*S, *Vgsc-*V402L, and *Vgsc-*I1527T^37^ or *Gste2-*L119V and *Gste2-*I114T^42,43^. These alleles have been detected at increasing prevalence across East, West, and Central African populations, suggesting adaptive gene flow and continentwide dissemination. Additionally, gene copy number amplification at loci such as the *Cyp6aa/p* gene cluster^5,44^, *Cyp6m2*^45^*, Coeae2f*^41^, and *Coeae2–7g*^40^ contributes to metabolic detoxification and cross-resistance to multiple classes of insecticides.

A recent genomic investigation of *An. gambiae* whole-genome data from western Ethiopia revealed high frequencies of known insecticide resistance markers accompanied by a signal of selection. These have also been previously reported from other East and Central African countries^23,37,41,46,47^. They included the non-synonymous substitutions *Vgsc-*L995F and *Vgsc-*L995S at the voltage-gated sodium channel (*Vgsc*) gene, which encodes the neuronal target of pyrethroids, and copy number variants at cytochrome *P450* loci (*Cyp6aa/p*, e.g., *Cyp6p4*, *Cyp6m2*), the detoxification gene *Cyp9k1*, and the glutathione S-transferase gene *Gste2*^23^. However, it is unknown whether these markers result from adaptive gene flow across the broader East and Central African regions or represent independent evolutionary events.

In this study, we analysed whole-genome sequence data of *An. gambiae* collected from western Ethiopia and compared them with populations from central and East Africa. Using analysis of population structure, we investigate whether Ethiopian *An. gambiae* are connected by gene flow to geographically nearby populations. We then investigate the adaptive allele sharing of insecticide resistance loci across the region.

## Materials and Methods

### Study sites and mosquito collection

This study examined 913 *Anopheles gambiae s. s*. mosquitoes that were whole-genome sequenced as part of the data available from the *Anopheles gambiae* 1000 Genomes (Ag1000G) project^7^. This included 79 samples collected in 2023 from western Ethiopia, specifically Benishangul-Gumuz and Gambella, during the major and minor malaria transmission seasons as described previously (Gemechu et al., 2026). We also included 50 *An. gambiae* from Kenya (AG1000G-KE, 1274-VO-KE-KAMAU-VMF00246); 252 from Uganda (1178-VO-UG-LAWNICZAK-VMF00025, AG1000G-UG); 291 from Tanzania (1246-VO-TZ-KABULA-VMF00197, AG1000G-TZ); and 241 from the Democratic Republic of Congo (1264-VO-CD-WATSENGA-VMF00164, AG1000G-CD) collected between 2000 and 2020, publicly available in the Vector Observatory (https://malariagen.github.io/vector-data/ag3/ag3.0.html) (Figure 1).

### Whole genome sequencing and data processing

DNA of each Anopheles mosquito was extracted using Qiagen DNeasy Blood and Tissue kit (Qiagen Sciences, MD, USA) according to manufacturer’s protocol. *Anopheles gambiae s.s*. specimens were whole-genome sequenced in accordance with the guidelines of Ag1000G^31^. Briefly, paired-end multiplex libraries were generated according to the Illumina protocol, with DNA fragmentation performed using Covaris Adaptive Focused Acoustics instead of nebulisation. Each multiplex contained 12 uniquely tagged individuals, and sequencing was performed on the Illumina HiSeq X platform to generate 150-bp paired-end reads.

Sequencing reads were mapped to the *AgamP4* reference genome of *Anopheles gambiae* using the BWA-mem (Burrows-Wheeler Aligner) algorithm^48–51^. Variant discovery followed the Ag1000G project framework^31^, with SNPs called using GATK v.3.7.0^52^. To ensure robust inference, samples with insufficient depth (<10X median coverage), poor genome representation (<50%), or elevated contamination (>4.5%) were excluded. High-quality biallelic SNPs were organised into haplotypes using a mix of read-backed phasing with WhatsHap v1.0^53^ and statistical phasing with SHAPEIT v4.2^54^. Copy number variants (CNVs) were identified using the Gaussian Hidden Markov Model approach^44^, leveraging normalised coverage profiles to estimate copy numbers across genomic windows. CNV calls with high likelihood scores (>1000) were retained, while individuals with excessive coverage variance were removed to minimise spurious signals. Full data processing pipelines are available via the MalariaGEN/pipelines GitHub repository^55^.

### Population connectivity and adaptive gene flow

To investigate the pattern of population connectivity, we constructed an unrooted neighbor-joining tree (NJT) with city-block distance and performed principal component analyses (PCA) across the 3L chromosome arm (15–41 Mbp), a genomic region minimally impacted by large structural variants such as inversions and deletions^7^. Analyses were based on 100,000 biallelic SNPs with a minimum allele count > 2 and no missing genotypes. Both NJT and PCA were implemented using the MalariaGen_data Python package https://malariagen.github.io/malariagen-data-python/latest/Ag3.html). To further corroborate the NJT and PCA, genetic differentiation was measured using pairwise Hudson’s *F*_*ST*_^56^. To ensure robust estimates, only cohorts with at least 10 samples were included. For the East and Central African cohorts, diversity statistics, including nucleotide diversity (θπ), Watterson’s theta (θw), and Tajima’s D, were calculated by sampling site and season to assess temporal variation. All diversity statistics were computed using the built-in functions of the malariagen_data Python package.

To investigate evidence for adaptive gene flow in *An. gambiae* across East-Central Africa, we performed diplotype clustering on loci previously identified as under selection in Ethiopia^23^. Hierarchical clustering was conducted using city-block genetic distance and complete linkage, implemented in the built-in malariagen_data Python function. The heatmap beneath the dendrogram represents individual heterozygosity, with darker shading indicating higher heterozygosity. Below this, CNV copy number is shown in light grey where absent. SNP tracks are also displayed: white and dark shading denote homozygous SNPs, and light grey indicates heterozygous SNPs.

## Results

### Genomic population structure

To investigate the genomic population structure of *Anopheles gambiae* in western Ethiopia, we applied a principal component analysis (PCA) and a neighbour-joining tree (NJT) using 100,000 SNPs from chromosome 3L, a region commonly used to investigate population connectivity because it is free from inversions^7,57^. PCA was used to visualize clustering patterns and relative differentiation among individuals, while the NJT provided a phylogenetic framework to assess relationships based on pairwise genetic distances. Using both approaches allowed us to cross-validate signals of population structure and ensure that subtle patterns of connectivity were not an artefact of a single method. The Ethiopian samples formed a single cluster on the PCA (Figure 2a) and NJT (Figure 2b), suggesting unrestricted gene flow in western Ethiopia. Furthermore, low genetic differentiation was observed among western Ethiopian populations, with *F*_*ST*_ values of 0.000 to 0.003.

To contextualise the geographic population structure of the Ethiopian populations, we then extended our analysis to include neighbouring East and Central African populations, including the Democratic Republic of the Congo, Kenya, Tanzania, and Uganda. The PCA revealed that the Ethiopian populations from Gambella and Benishangul-Gumuz clustered close to neighbouring East and Central African populations, including the eastern and western regions of Uganda, Turkana and Busia in the northwestern regions of Kenya, Kagera in northern Tanzania, and Nord-Ubangi in northwestern DRC. In the full PCA, these populations formed a broad inland East African grouping rather than sharply defined clusters. To examine fine-scale variation within this grouping, we restricted the PCA to these cohorts only (Figure S2). Under these restricted view, Ethiopian populations (Benishangul-Gumuz and Gambella) and Nord-Ubangi (northern DRC) separated slightly from one another and from Kenya and Tanzania cohorts, indicating subtle differentiation despite their overall genetic similarity to inland East Africa populations. This trend was supported by pairwise *F*_*ST*_ estimates. Ethiopian and Nord-Ubangi populations showed a higher *F*_*ST*_ range (0.003–0.01) in comparison to populations in Uganda, northwestern Kenya, and northern Tanzania with a lower *F*_*ST*_ value (0.000 to 0.004). These values indicate that, while Ethiopia and northern DRC remain broadly connected to inland East Africa, they carry subtle genetic differentiations relative to their neighbours. In the PCA and NJT of all populations, we also observed that Kongo-Central formed a separate cluster and had a relatively high *F*_*ST*_ measure of genetic differentiation (0.014–0.023) in comparison to all other East and Central African populations. Central Kasai and Haut-Katanga in the central and southern DRC also formed a separate cluster together and differentiated from other eastern and central African populations with an *F*_*ST*_ of 0.011–0.032. Finally, the coastal populations of Kilifi (Kenya) and Tanga (Tanzania) formed a distinct PCA and NJT cluster together, which differentiated from other East and Central African populations with *F*_*ST*_ values of 0.047 to 0.074 (Figure S4).

### Genetic diversity

Genomic diversity was assessed to provide insights in to the demographic history, population connectivity, and adaptive potential of *Anopheles gambiae* across East and Central Africa. To capture these patterns, we examined the genetic diversity statistics nucleotide diversity (θπ), Watterson’s θ_w_, and Tajima’s D across cohorts from East and Central Africa with a minimum cohort size of 10 individuals. Ethiopian populations from Gambella and Benishangul-Gumuz had nucleotide diversity (θπ) values ranging from ~0.018 to 0.022 and Watterson's theta (θ_w_) values of ~0.033 to 0.035. These values were broadly comparable to those observed in other nearby inland East African populations, including *An. gambiae* from western and eastern Uganda (θπ=~0.025–0.026; θ_w=_ 0.035–0.36), Kagera in Tanzania (θπ=~0.026–0.106; θ_w=_ 0.034–0.111), Busia in Kenya (θπ=~0.026–0.028; θ_w=_~0.036–0.04), and Nord-Ubangi in northern DRC (θπ=0.027, θ_w=_ 0.036). Tajima’s D was more varied across the populations but similar, with values ranging from (D = −1.27). An exception was a cohort sampled during the dry season from Kagera, Tanzania, in February 2019, which had a higher nucleotide diversity (θπ = 0.11) and lower Tajima’s D (D = −0.2). A collection from the intermittent period before the dry season from Busia, Kenya, in July also showed slightly higher genetic diversity (θπ = 0.03) and a lower Tajima's D value (D ≈ −1.27), suggesting population expansion at the time these cohorts were sampled.

Compared with the Ethiopian, inland Tanzanian (Kagera, Tanga), inland Kenyan (Kilifi, Busia), and northern DRC (Nord-Ubangi) cohorts, the southern and central DRC populations, including Kongo-Central and Central Kasai, showed a comparatively lower nucleotide diversity (θπ = 0.025) and Watterson’s estimator (θ_w_ = 0.03), together with less negative Tajima’s D values (D = −0.71). Findings indicate a smaller effective population size, consistent with restricted gene flow between these populations and the wider East African region, as inferred from population structure analysis. Coastal populations from Kilifi (Kenya) and Tanga (Tanzania) had the lowest genetic diversity (θπ = 0.022; θ_w_ = 0.023 and θπ = 0.022; θ_w_ = 0.023, respectively) and the least negative Tajima’s D-values (D = −0.091 and D = −0.11, respectively), indicating a population bottleneck in the coastal East African *An. gambiae* as observed previously (Figure 4)^58^.

### Adaptive gene flow

To investigate evidence for adaptive gene flow in *An. gambiae* across East and Central Africa, we performed diplotype clustering on loci previously identified as under selection in Ethiopia^23^. First, we investigated the voltage-gated sodium channel (*Vgsc*) gene (2L: 2,350,000–2,450,000), which is linked to target-site pyrethroid resistance^59^. The diplotype clustering plot revealed three low-heterozygosity clusters, indicating variation potentially under selection (Figure 4). **Cluster A** consisted of samples from Kenya (Busia/Teso North), Tanzania (Kagera/Muleba), and Ethiopia (Benishangul-Gumuz and Gambella). It was characterised by the *Vgsc*-L995S allele, which is common in East Africa and linked to insecticide resistance^16,23,60,61^. Individuals in **Cluster B** also had the L995S alleles but comprised only samples from Kenya and Tanzania (Figure 4), suggesting the presence of two independent evolutionary backgrounds in East Africa. In contrast, **Cluster C**, including *An. gambiae* from Ethiopia (Benishangul-Gumuz and Gambella) and the DRC (Central Kasai, Haut-Katanga, Kongo-Central, and Nord-Ubangi), shared the L995F allele in combination with R254K. Although L995F, which is common in West Africa, is known to confer resistance to pyrethroids^37^, the association of R254K has not been previously described. Overall, results reveal that *An. gambiae* from Ethiopia shares adaptive gene flow for the L995S allele with East African populations and for the L995F allele with Central African populations (Figure 5).

Within the *Cyp6aa/p* gene cluster, we examined copy-number variation across the whole cluster and nonsynonymous substitutions within *Cyp6p4* because the I236M SNP in this gene has been associated with pyrethroid resistance^5^. The clustering analysis revealed three major clusters with low heterozygosity. The first low-heterozygosity cluster (**Cluster A**) is associated with both CNVs at the Cyp6aa1 gene, ranging from 4 to 12 copies and the *Cyp6p4*-I333V SNP. The second cluster (**Cluster B**) had no clear association with CNVs or SNPs. The third cluster (**Cluster C**), however, comprised individuals from across the region, including Ethiopia (Benishangul-Gumuz and Gambella), Kenya (Busia, Kiambu, and Turkana), Uganda (eastern and western regions), Tanzania (Kagera), and the Democratic Republic of Congo (Nord-Ubangi). All individuals within the cluster had the I236M substitution associated with insecticide resistance^5^. Although we also observed some individuals from Tanzania which carried the I236M allele outside of the cluster, the substitution was generally associated with CNVs with a shared evolutionary background across East Africa and the northern DRC (Figure 6).

The diplotype clustering of the *Cyp9k1* gene region (X:15,240,572–15,242,862) revealed two major low-heterozygosity clusters (Figure 6). **Cluster A** comprised *An. gambiae* samples exclusively from Kongo-Central (DRC), characterised by an association with copy-number variation (CNV) ranging between four and five copies. **Cluster B** included populations from Ethiopia (Benishangul-Gumuz and Gambella), Uganda (eastern and western); Tanzania (Kagera); Kenya (Busia); and Nord-Ubangi (DRC) and also included individuals with CNVs ranging between one and five copies. Findings suggest a shared evolutionary origin of *Cyp9k1* CNVs across East Africa and northern DRC but a unique diplotype background in central DRC (Figure 7).

The diplotype clustering of the *Gste2* gene region (3R:28,590,243–28,602,280) revealed two low-heterozygosity clusters with a relatively high number of individuals greater than 20 (Figure 8). Two major clusters were present. **Cluster A** comprised individuals from Tanga (Tanzania), Kilifi (Kenya), and Haut-Katanga (DRC). Although characterised by the substitution *K92R,* individuals within this cluster did not have CNVs or a SNP previously associated with insecticide resistance, and therefore, it is unclear whether individuals within this cluster are under selection. **Cluster B** included *An. gambiae* from eastern and western Uganda, Kagera (Tanzania), Busia (Kenya), and Gambella (Ethiopia). However, once again, there were no CNVs or SNPs associated with the cluster and no clear link to a metabolic resistance marker. The lack of low homozygosity variation associated with insecticides is not unexpected, given that only a small and indistinct selection peak at *Gste2* was previously observed in the Ethiopian cohorts^23^ (Figure 8).

## Discussion

Our findings demonstrate that *An. gambiae* populations across East Africa exhibited broad regional connectivity, including western Ethiopia, inland Kenya, inland Tanzania, Uganda, and the northern DRC. This pattern is consistent with recent genomic evidence showing high effective migration rates across East-Central inland populations, potentially driven by similar environmental conditions^9^. Importantly, the connectivity we observed likely reflects the ecology of *An. gambiae*, which favours open, humid savanna and peri-forest habitats with abundant temporary pools that are widely distributed over the inland landscapes.

However, within this highly connected region, we also found evidence of subtle geographical population structure between western Ethiopian populations and Nord-Ubangi in the northern DRC. This differentiation may be promoted by the intervening East African arid valley floors and steep escarpments, as well as montane ridges, which interrupt the continuity of suitable breeding habitats. In western Ethiopia, expansive lowland floodplains provide seasonal pools that sustain *An. gambiae;* the rifts’ dry basin and rugged terrain reduce habitat availability and constrain dispersal corridors toward central Africa. These ecological discontinuities likely limit effective migration and contribute to the observed genetic divergence between Ethiopian and northern DRC populations^1,62^. We also observed evidence of restricted gene flow between the wider eastern and northern DRC regions and *An. gambiae* from other locations in Central Africa, including Central Kasai and southern Haut-Katanga. Nord-Ubangi in the northern DRC lies at the northern fringe of the Congo Basin tropical rainforest, forming a transition zone between dense forest and open savanna. Central Kasai itself lies within the dense Congo Basin rainforest, which may restrict mosquito dispersal due to deep canopy shade that blocks sunlight, the absence of breeding pools for larval development, and the lack of human hosts in the interior forest^63–65^. Haut-Katanga is characterised by plateau and savanna ecosystems where arid conditions may also restrict gene flow by imposing severe desiccation stress on adults and limiting larval development to short-lived habitats^7,11^.

In addition to a lower population size due to geographic isolation, a population experiencing more extreme environmental conditions may be affected by temporal bottlenecks that promote genetic differentiation relative to more stable populations^9^. We found slightly lower genetic diversity and less negative Tajima’s D in both Haut-Katanga and Central Kasai compared to the wider, connected region, suggesting that populations in south-central DRC may experience a different demography. We further found restricted gene flow between inland East Africa and the coastal populations of Kenya and Tanzania. Unlike inland East Africa, where perennial wetland and riverine systems sustain large, interconnected populations, coastal habitats are fragmented and ephemeral, drying outside the rainy season^11,58^. These ecological conditions may reduce effective population size and amplify genetic drift to promote population structure^1,9,58,66^.

In addition to landscape and environmental barriers, targeted vector control interventions such as ITNs and IRS can induce severe population bottlenecks and genetic differentiation^10,43^. Across East and Central Africa, these interventions are widely implemented^10,67^ creating intense selection pressures^58^. However, deployment is not uniform, as IRS campaigns are concentrated in high-burden zones such as coastal Kenya^68^ and Tanzania^69^, as well as southern DRC^70^. At the same time, inland East Africa and northern DRC rely more heavily on ITN. Notably, Kilifi region on the Kenyan country coast also received an ITN campaign during the sampling period, supplementing IRS efforts in coastal zones^71^. This heterogeneity suggests that intervention intensity may contribute to macrogeographic population structure, potentially acting synergistically with ecological barriers to exacerbate bottlenecks in naturally isolated or unstable habitats, but it requires further investigation, including a more detailed assessment of differences in insecticide-based control measures.

Our investigation of adaptive allele sharing across East-Central Africa showed a locus-specific pattern in the relationship between genome-wide population structure and adaptive gene flow. At the *Cyp6aa/p* gene cluster and *Cyp9k1,* CNV variant sharing identified through diplotype clustering broadly mirrored genome-wide population connectivity, with inland cohorts exhibiting a common cluster consistent with their connectivity. In comparison, the isolated populations, such as Haut-Katanga and Central Kasai in the DRC, carried distinct CNV-associated clusters independent of the wider inland group. Amplifications at *Cyp6aa/p* and *Cyp9k1* are well-established drivers of metabolic resistance, enhancing detoxification of pyrethroids and contributing to widespread resistance phenotypes^5,15,44,72^. Their broad dissemination across inland East-Central Africa likely reflects both selection pressure from long-term pyrethroid use and the capacity of these CNVs to spread rapidly through connected populations^7,9^. The concordance between *Cyp6aa/p* variant sharing and genome-wide connectivity suggests that metabolic resistance loci can diffuse efficiently across regions where ecological continuity sustains large, interconnected mosquito populations, reinforcing the need for regional surveillance and harmonised insecticide resistance management strategies.

In contrast, at the *Vgsc* locus, the L995F variant was restricted to Ethiopia and the DRC, despite population connectivity with inland East Africa. L995F, the canonical ‘kdr-west’ mutation, has historically been associated with West and Central African populations^36,37,59^. Its presence in Ethiopia likely reflects long-range gene flow along the Nile-Congo corridor, while its absence in Kenya, Tanzania, and Uganda underscores the dominance of the alternative L995S (kdr-east) mutation in East Africa^73,74^. This discordance suggests that adaptive allele dissemination can diverge from genome-wide connectivity. In this case, patterns may be shaped by locus-specific selection pressures, ecological contexts, and historical introduction events^1^, which should be considered when interpreting resistance evolution.

## Conclusion

This study demonstrates that adaptive allele sharing in *Anopheles gambiae* across East-Central Africa is locus-specific, with metabolic resistance variants broadly mirroring genome-wide connectivity, while target-site mutations remain geographically restricted. Therefore, resistance evolution in *An. gambiae* must be addressed through strategies that anticipate locus-specific dynamics. Importantly, Ethiopia emerges as a genomic “melting pot”, where *Vgsc*-L995F/S alleles from Central and East Africa converge, creating a critical zone for monitoring emergent resistance loci and their interception. This convergence highlights Ethiopia as an important area for regional policy coordination, since variants introduced here may have the potential to spread across wider inland Africa.

Taken together, these contrasting scenarios suggest that effective resistance management requires a dual operational strategy: coordinated cross-border monitoring and harmonised insecticide intervention policies to tackle the risk of rapid resistance allele dissemination across highly connected populations. This is combined with site-specific interventions tailored to ecological isolates such as the Congo Basin rainforests and Haut-Katanga and coastal East Africa populations, where divergent resistance trajectories are likely to emerge. Such an integrated approach combining regional coordination with locally tailored strategies will be critical to sustain malaria control in East-Central Africa and prevent adaptive alleles from undermining intervention effectiveness. Future actions should prioritise integrating genomic surveillance into routine vector control programmes, strengthening cross-border collaboration, and developing adaptive resistance management strategies that respond to both regional connectivity and local divergence.

## Supplementary Material

1

## Figures and Tables

**Figure 1.**
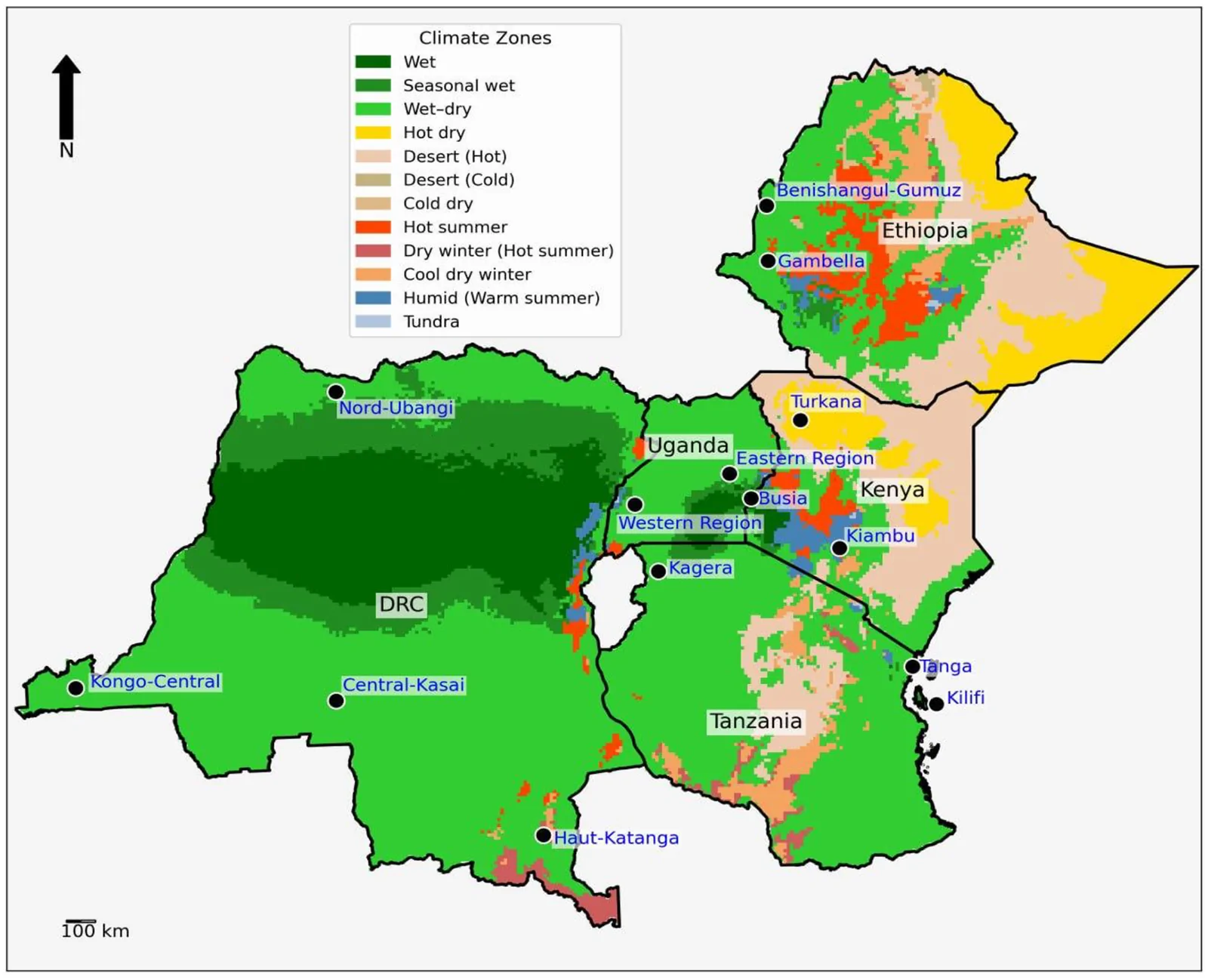
Distribution of study sites across Ethiopia, Uganda, Kenya, Tanzania, and the Democratic Republic of the Congo, overlaid with Koppen climate zones.

**Figure 2.**
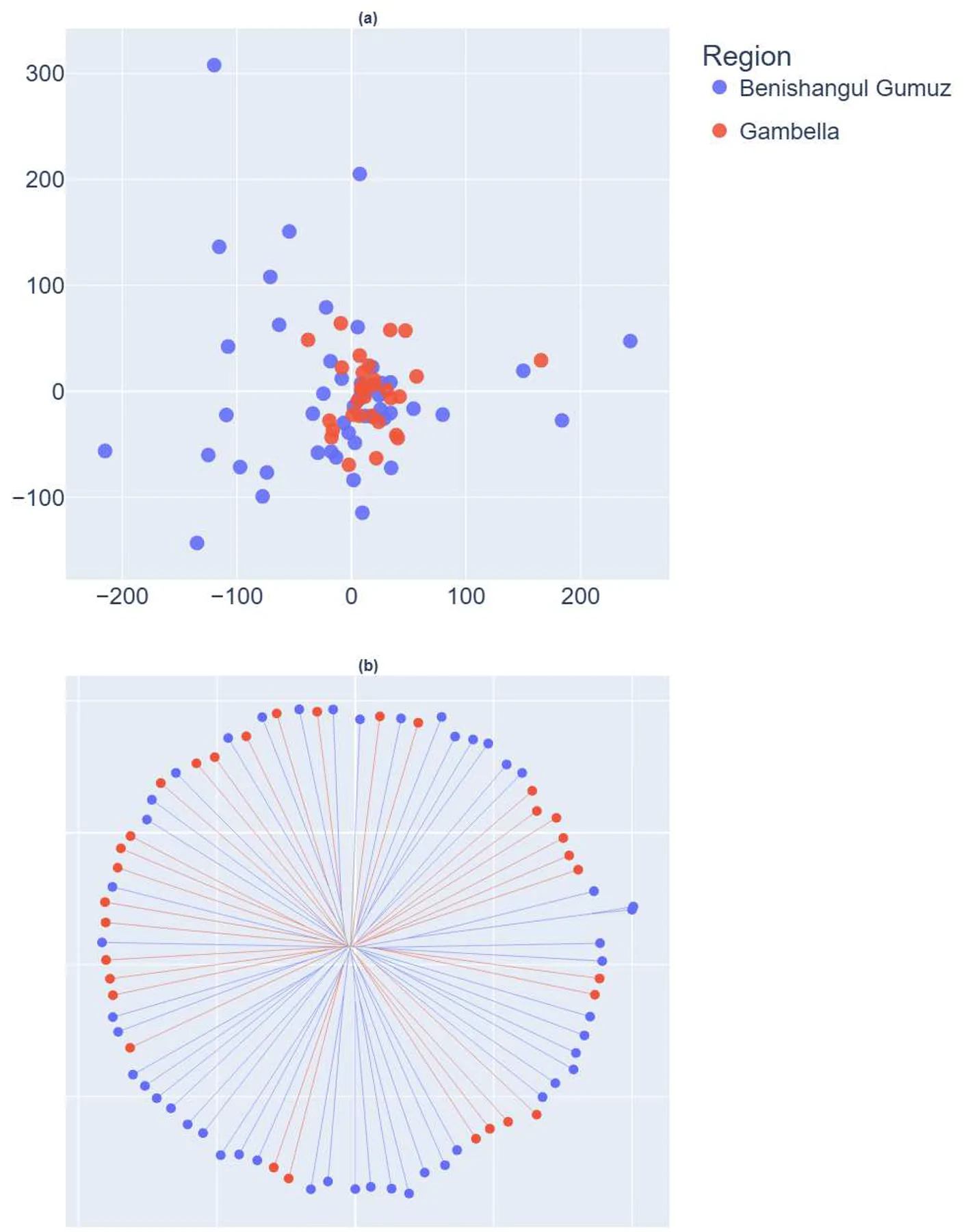
Principal component analysis (PCA) (a) and neighbour-joining tree (b) of *Anopheles gambiae s.s*. population structure for Ethiopian samples from the Benishangul-Gumuz and Gambella regions. Each point represents an individual sample and points colour represents collection site.

**Figure 3.**
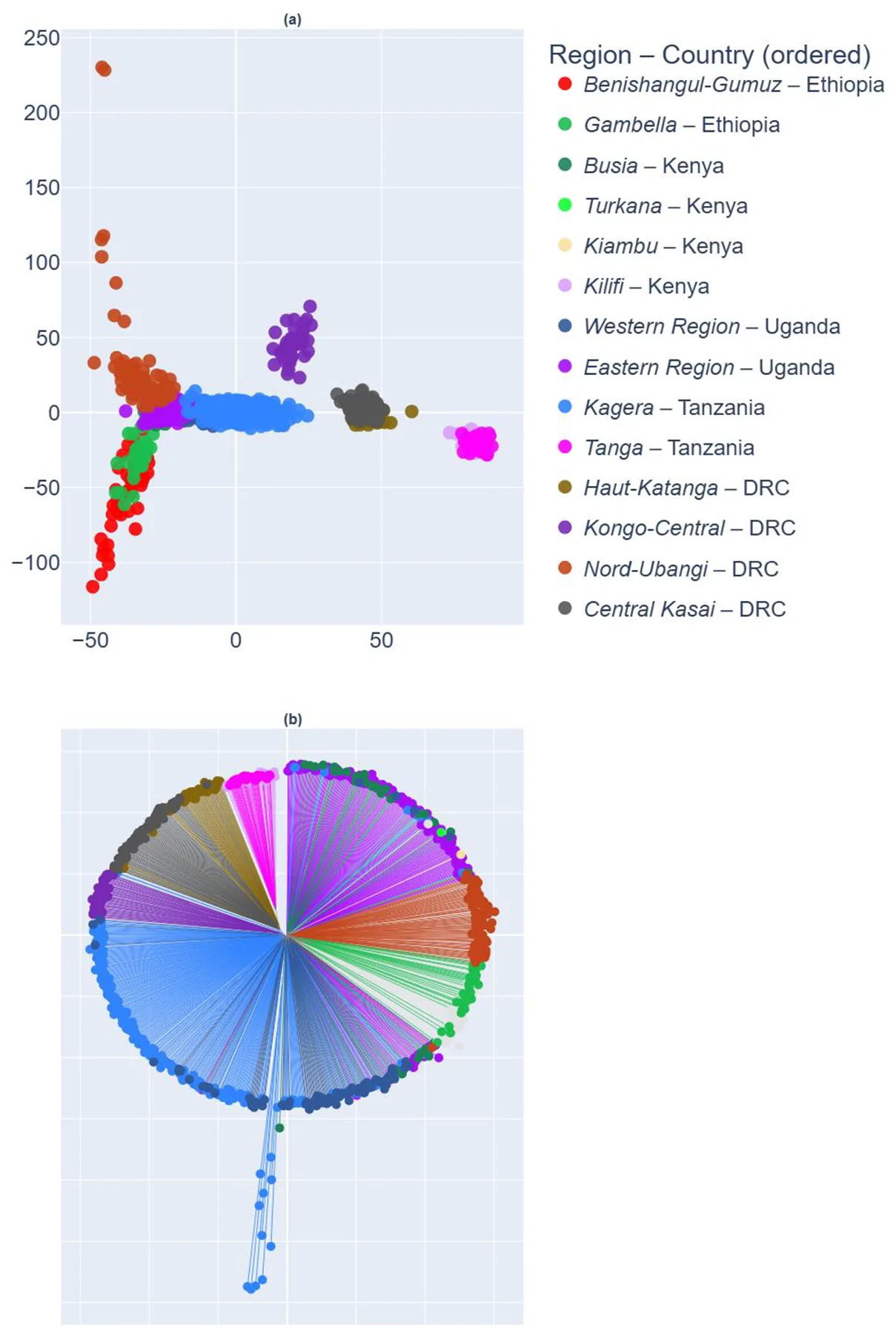
Principal component analysis (PCA) (a) and neighbor-joining tree (NJT) (b) of *Anopheles gambiae s.s*. population structure for East and Central African samples. Each point represents an individual sample and points colour represents collection site.

**Figure 4.**
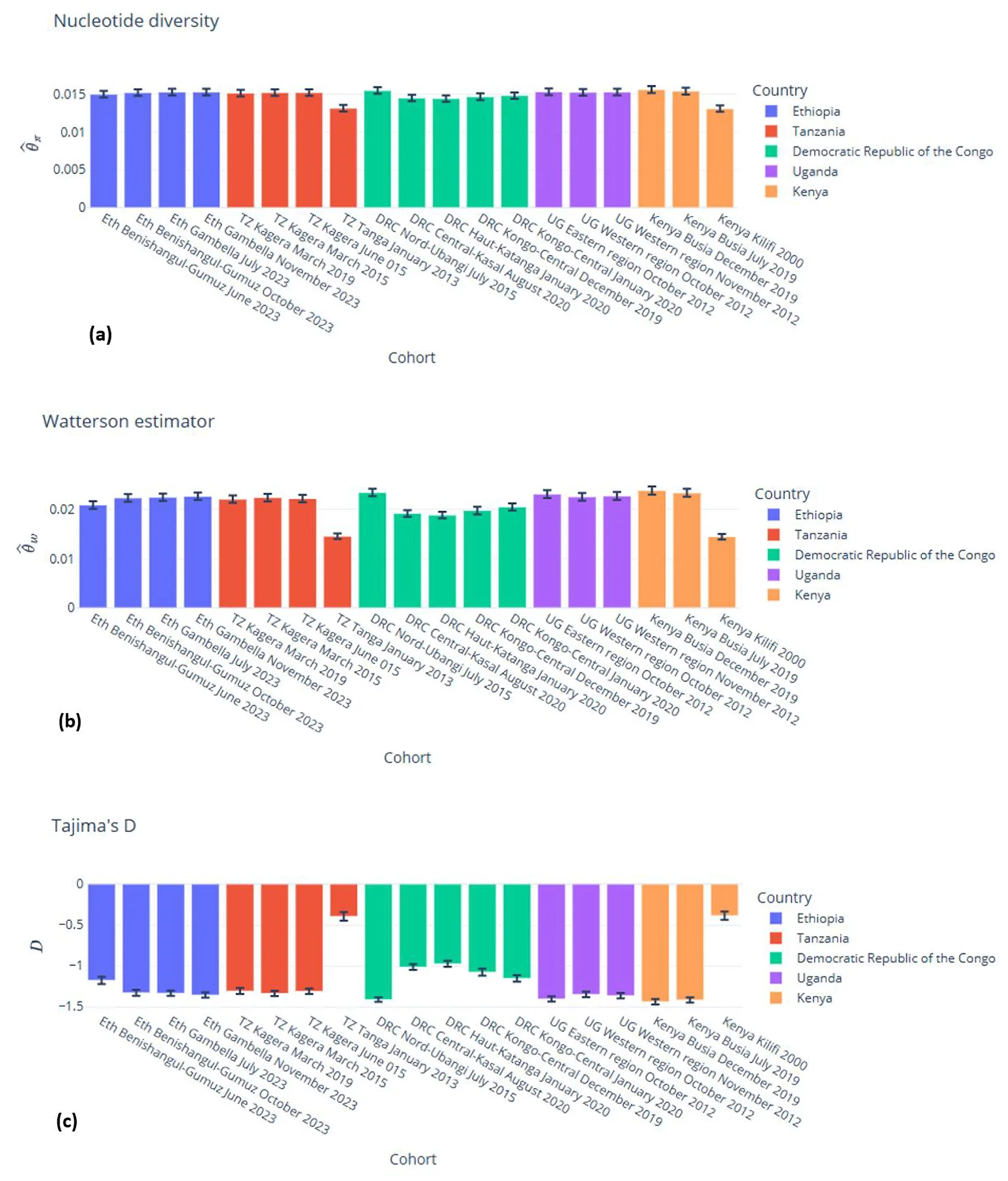
Plots of the genetic diversity statistics (a) nucleotide diversity, (b) Watterson’s estimator, and (c) Tajima’s D of *Anopheles gambiae s.s*. populations across East and Central African countries, organised by month and collection location.

**Figure 5.**
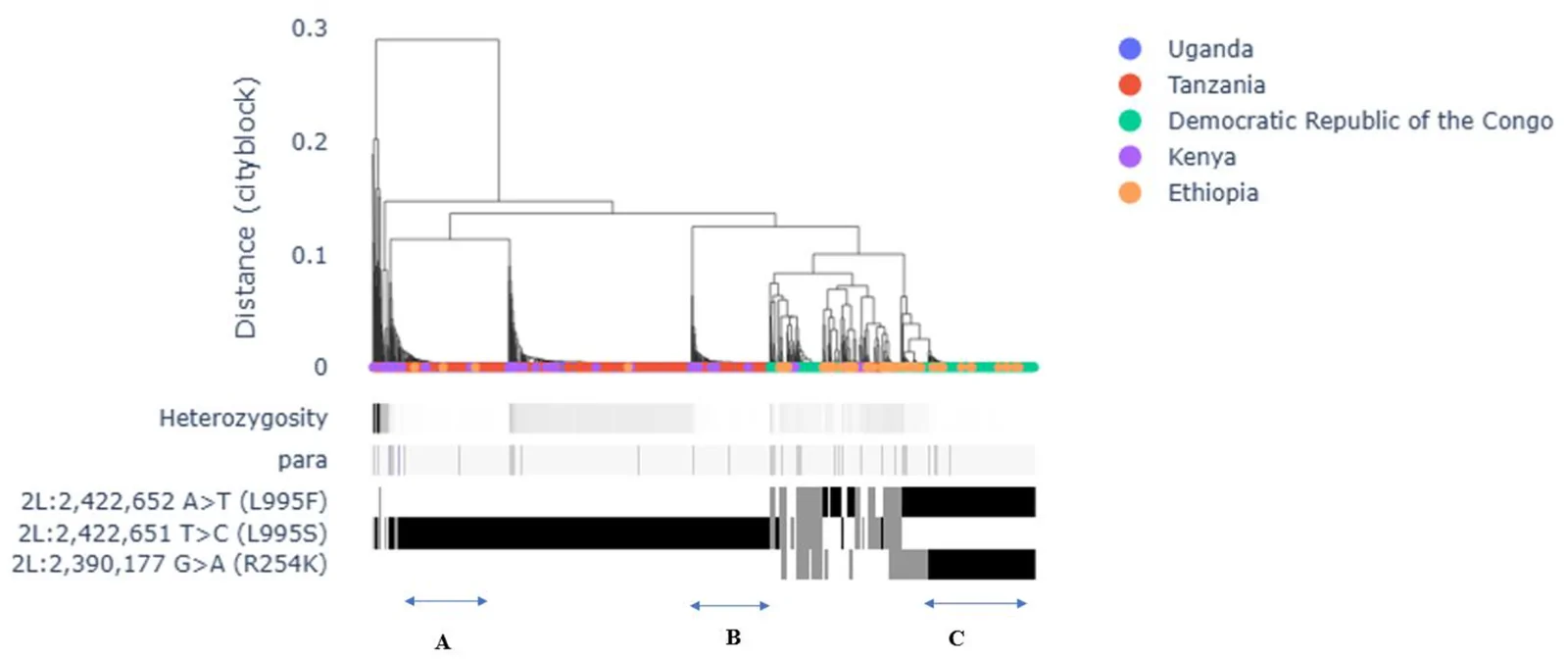
Diplotype clustering of *Anopheles gambiae* samples at the *Vgsc* locus for populations across East and Central Africa. The heatmap underneath the dendrogram represents individual heterozygosity, with darker shading indicating higher heterozygosity. Below the heterozygosity layer, CNV copy number is indicated by light shades of grey, denoting its absence. SNP trackers are also displayed: white and dark shading denote homozygous SNPs, and light grey indicates heterozygous SNPs.

**Figure 6.**
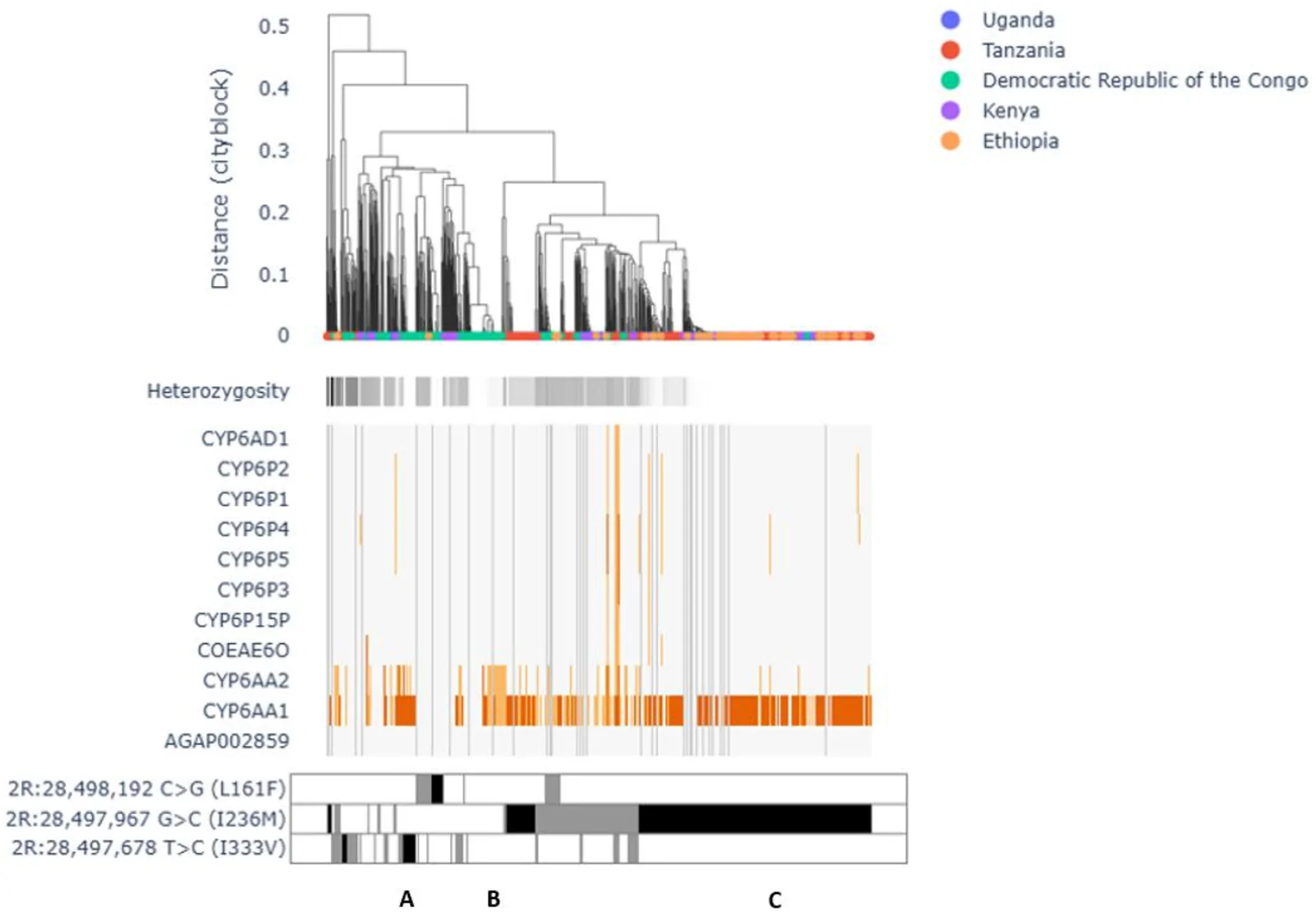
Diplotype clustering of *Anopheles gambiae* samples from Uganda, Tanzania, the Democratic Republic of the Congo (DRC), Kenya, and Ethiopia within the whole *Cyp6* gene cluster and the *Cyp6p4* gene locus on chromosome 2R contig. The plot shows diploidy relationships. The heatmap beneath represents individual heterozygosity (darker shading = higher heterozygosity). CNV copy number is indicated below, with light grey denoting absence. SNP tracks are also displayed: white and dark shading denote homozygous SNPs, while light grey indicates heterozygous SNPs.

**Figure 7.**
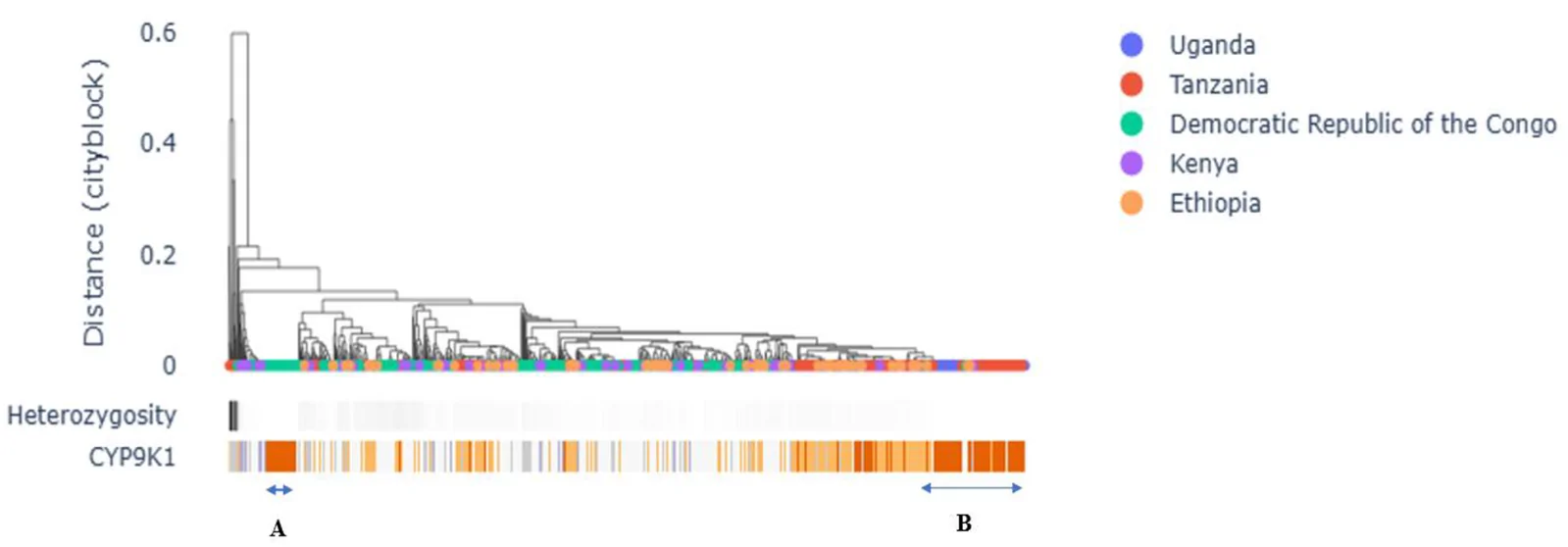
Diplotype clustering of *Anopheles gambiae* samples from Uganda, Tanzania, the Democratic Republic of the Congo (DRC), Kenya, and Ethiopia within the *Cyp9k1* gene locus on chromosome X. The heatmap beneath represents individual heterozygosity (darker shading = higher heterozygosity). CNV copy number is indicated below, with deep orange denoting selection for the CNV.

**Figure 9.**
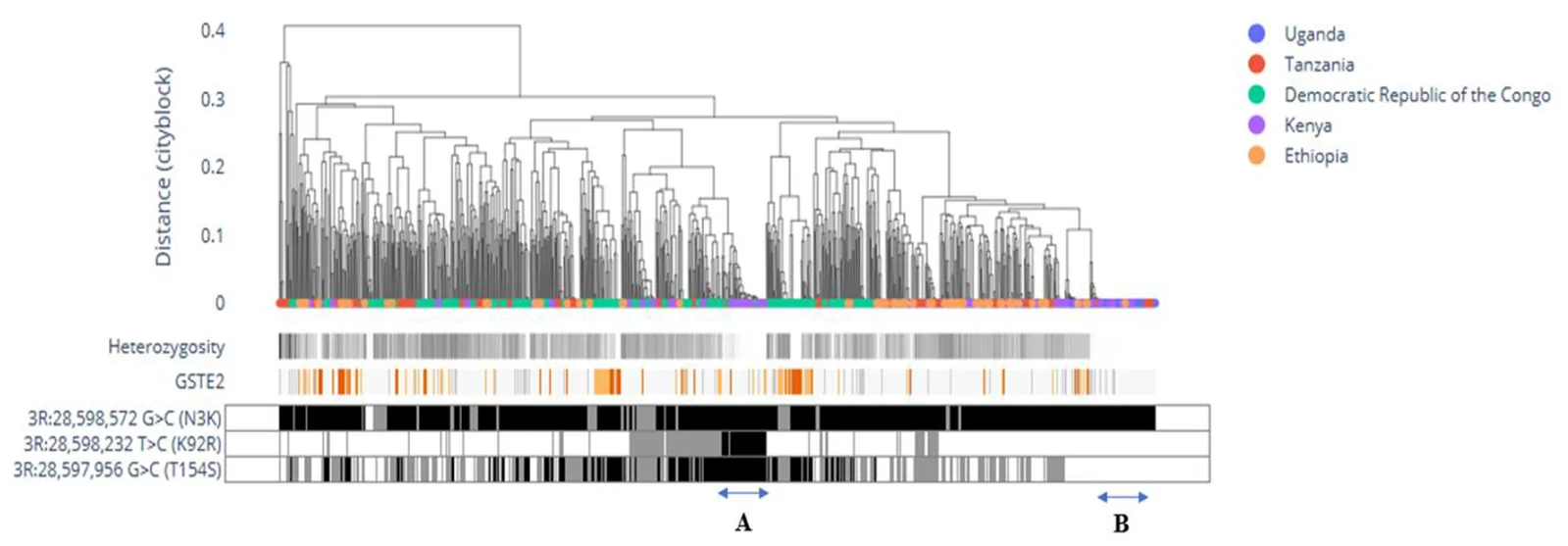
Diplotype clustering of *Anopheles gambiae* samples from Uganda, Tanzania, the Democratic Republic of the Congo (DRC), Kenya, and Ethiopia within the *Gste2* gene locus on chromosome 3R. The heatmap beneath represents individual heterozygosity (darker shading = higher heterozygosity). CNV copy number is indicated below, with light grey denoting absence. SNP tracks are also displayed: white and dark shading denote homozygous SNPs, while light grey indicates heterozygous SNPs.

## Data Availability

The sequences of the samples identified in this study were submitted to the European Nucleotide Archive (ENA; accession numbers in Table S2).

## Abbreviations

CNV: Copy Number Variants
DRC: Democratic Republic of the Congo
ENA: European Nucleotide Archive
*Gste2*: Glutathione S-transferase epsilon class-2 genes
GWSS: genome-wide selection scan
SNP: Single Nucleotide Polymorphisms
*Vgsc*: Voltage-gated Sodium Channel
WGS: Whole-genome sequencing

## References

[1] Hemming-Schroeder E. Ecological drivers of genetic connectivity for African malaria vectors Anopheles gambiae and An. arabiensis. Sci. Rep. 10, 19946 (2020). 10.1038/s41598-020-76248-2 PMC7673128 33203917

[2] Saucedo O. & Tien J. H. Host movement, transmission hot spots, and vector-borne disease dynamics on spatial networks. Infect. Dis. Model. 7, 742–760 (2022). 10.1016/j.idm.2022.10.006 PMC9672958 36439402

[3] Miller N. J. & Sappington T. W. ScienceDirect Role of dispersal in resistance evolution and spread. Curr. Opin. Insect Sci. 22, 1–7 (2017). 10.1016/j.cois.2017.04.005 28822491

[4] Schmidt The Population Genomics of Aedes aegypti: Progress and Prospects. Popul. Genomics Insects 201–221 (2021) doi:10.1007/13836_2021_93.

[5] Njoroge H. Identification of a rapidly-spreading triple mutant for high-level metabolic insecticide resistance in Anopheles gambiae provides a real-time molecular diagnostic for antimalarial intervention deployment. Mol. Ecol. 31, 4307–4318 (2022). 10.1111/mec.16591 PMC9424592 35775282

[6] Hanemaaijer M. J. Introgression between Anopheles gambiae and Anopheles coluzzii in Burkina Faso and its associations with kdr resistance and Plasmodium infection. Malar. J. 18, 127 (2019). 10.1186/s12936-019-2759-1 PMC6458625 30971230

[7] The Anopheles gambiae 1000 Genomes Consortium. Genetic diversity of the African malaria vector anopheles gambiae. Nature 552, 96–100 (2017). 10.1038/nature24995 PMC6026373 29186111

[8] Lucas E. R. A high throughput multi-locus insecticide resistance marker panel for tracking resistance emergence and spread in Anopheles gambiae. Sci. Rep. 9, 13335 (2019). 10.1038/s41598-019-49892-6 PMC6746726 31527637

[9] McCann R. S. Variation in spatial population structure in the Anopheles gambiae species complex. bioRxiv Prepr. Serv. Biol. (2024) doi:10.1101/2024.05.26.595955.

[10] Oxborough R. M., Chilito K. L. F., Tokponnon F. & Messenger L. A. Personal View Malaria vector control in sub-Saharan Africa : complex trade-offs to combat the growing threat of insecticide resistance. Lancet Planet. Heal. 8, e804–e812 (2024). 10.1016/S2542-5196(24)00172-4 39393381

[11] Pinto J. Geographic population structure of the African malaria vector Anopheles gambiae suggests a role for the forest-savannah biome transition as a barrier to gene flow. Evol. Appl. 6, 910–924 (2013). 10.1111/eva.12075 PMC3779092 24062800

[12] Sinka M. E. The dominant Anopheles vectors of human malaria in Africa, Europe and the Middle East: Occurrence data, distribution maps and bionomic précis. Parasites and Vectors 3, (2010). 10.1186/1756-3305-3-117 PMC3016360 21129198

[13] Kweyamba P. A. Contrasting vector competence of three main East African Anopheles malaria vector mosquitoes for Plasmodium falciparum. Sci. Rep. 15, 2286 (2025). 10.1038/s41598-025-86409-w PMC11747171 39833263

[14] Kamdem C. Anthropogenic Habitat Disturbance and Ecological Divergence between Incipient Species of the Malaria Mosquito Anophelesgambiae. PLoS One 7, e39453. (2012). 10.1371/journal.pone.0039453 PMC3382172 22745756

[15] Weetman D. Candidate-gene based GWAS identifies reproducible DNA markers for metabolic pyrethroid resistance from standing genetic variation in East African Anopheles gambiae. Sci. Rep. 2920 (2018) doi:10.1038/s41598-018-21265-5. PMC5811533 29440767

[16] Riveron J. M. Multiple insecticide resistance in the major malaria vector Anopheles funestus in southern Ghana : implications for malaria control. Parasit. Vectors 9, 504 (2016). 10.1186/s13071-016-1787-8 PMC5024453 27628765

[17] Eligo N. Anopheles arabiensis continues to be the primary vector of Plasmodium falciparum after decades of malaria control in southwestern Ethiopia. Malar. J. 23, 14 (2024). 10.1186/s12936-024-04840-2 PMC10777518 38195563

[18] Tsegaye A., Demissew A., Hawaria D., Getachew H. & Habtamu K. Susceptibility of primary, secondary and suspected vectors to Plasmodium vivax and Plasmodium falciparum infection in Ethiopia. Parasit. Vectors 15, 384 (2022). 10.1186/s13071-022-05467-5 PMC9587640 36271436

[19] Woyessa D. & Yewhalaw D. Correction to: Anopheles mosquito fauna, blood meal sources and transmission intensity from high and moderate malaria endemic areas of Ethiopia (Scientific Reports, (2025), 15, 1, (10636), 10.1038/s41598-025-94739-y). Sci. Rep. 15, 10636 (2025). PMC11950359 40148450

[20] Pendy N. M. L. Ecological plasticity to ions concentration determines genetic response and dominance of Anopheles coluzzii larvae in urban coastal habitats of Central Africa. Sci. Rep. 11, 15781 (2021). 10.1038/s41598-021-94258-6 PMC8338965 34349141

[21] Akpodji S., Agbangla C., Padonou G. G., Affolabi Z. K. & Koukpo Z. C. Spatial and temporal distribution of four malaria vector species and their relative contributions to Plasmodium falciparum transmission along the south – north transect of Benin, West Africa. Trop. Med. Health 53, 147 (2025). 10.1186/s41182-025-00822-5 PMC12573912 41168853

[22] Amoako E. K. Genomic population structure and insecticide resistance mechanisms in the malaria vector An. coluzzii across contrasting bioclimatic zones in West Africa. BMC Genomics 27, 140 (2026). 10.1186/s12864-025-12508-7 PMC12870732 41501650

[23] Gemechu F., Bennett K. L., Hernandez-koutoucheva A., Miles A. & Clarkson C. S. Genomic analysis reveals selection pressure and the emergence of molecular insecticide resistance in the malaria vector, Anopheles gambiae, from Western. Parasit. Vectors (2026). 10.1186/s13071-026-07582-z PMC13726317 42972566

[24] Kinfe E., Tekie H., Nigatu W., Abate E. & Kifle T. First report of African malaria vector Anopheles gambiae (Diptera : Culicidae) from Ethiopia. Ethiop. j. public Heal. nutr. 3, 23–33 (2012).

[25] Appawu M. Malaria transmission dynamics at a site in northern Ghana proposed for testing malaria vaccines. Trop. Med. Int. Heal. 9, 164–170 (2004). 10.1046/j.1365-3156.2003.01162.x 14728621

[26] Giraldo D. & McMeniman C. J. Quantifying Mosquito Host Preference. Cold Spring Harb. Protoc. 2024, 107663 (2024). 10.1101/pdb.top107663 37612146

[27] Gimonneau G. A behavioral mechanism underlying ecological divergence in the malaria mosquito Anopheles gambiae. Behav. Ecol. 21, 1087–1092 (2010). 10.1093/beheco/arq114 PMC2920295 22476108

[28] Wamae P. M., Githeko A. K., Otieno G. O., Kabiru E. W. & Duombia S. O. Acta Tropica Early biting of the Anopheles gambiae s. s. and its challenges to vector control using insecticide treated nets in western Kenya highlands. Acta Trop. 150, 136–142 (2015). 10.1016/j.actatropica.2015.07.008 26209103

[29] Charlwood , 2018. Studies on the resting behaviour and host choice of Anopheles gambiae and An. arabiensis from Muleba, Tanzania. Med. Vet. Entomol. 32, 263–270 (2018). 10.1111/mve.12299 29479733

[30] Woyessa D. Species composition, infection rate and detection of resistant alleles in Anopheles funestus (Diptera : Culicidae) from Lare, a malaria hotspot district of Ethiopia. Malar. J. 22, 233 (2023). 10.1186/s12936-023-04667-3 PMC10422748 37573300

[31] The Anopheles gambiae 1000 Genomes Consortium. Genome variation and population structure among 1142 mosquitoes of the African malaria vector species Anopheles gambiae and Anopheles coluzzii. Genome Res. 30, 1533–1546 (2020). 10.1101/gr.262790.120 PMC7605271 32989001

[32] Mwima R. Assessing the population genetic structure and demographic history of Anopheles gambiae and Anopheles arabiensis at island and mainland sites in Uganda : implications for testing novel malaria vector control approaches. Malar. J. 25, 96 (2026). 10.1186/s12936-025-05768-x PMC12903332 41559777

[33] Okia M. Insecticide resistance status of the malaria mosquitoes : Anopheles gambiae and Anopheles funestus in eastern and northern Uganda. Malar. J. 17, 157 (2018). 10.1186/s12936-018-2293-6 PMC5889576 29625585

[34] Ramaita E. Current Research in Parasitology & Vector-Borne Diseases Insecticide resistance intensity in Anopheles gambiae (s. l .) from five malaria epidemiological zones in Kenya. Curr. Res. Parasitol. Vector-Borne Dis. 7, 100252 (2025). 10.1016/j.crpvbd.2025.100252 PMC11981760 40206848

[35] Wolie R. Z., Koffi A. A., Chabi J. & Alou L. P. A. Insecticide resistance and species diversity in Anopheles gambiae s. l. in Côte d ’ Ivoire. PLoS One 20, 1–12 (2025). 10.1371/journal.pone.0332497 PMC12483214 41026751

[36] Martinez-Torres. Molecular characterization of pyrethroid knockdown resistance (kdr) in the major malaria vector Anopheles gambiae s. s. Insect Mol. Biol. 7, 179–184 (1998). 10.1046/j.1365-2583.1998.72062.x 9535162

[37] Clarkson C. S. The genetic architecture of target- - site resistance to pyrethroid insecticides in the African malaria vectors Anopheles gambiae and Anopheles coluzzii. Mol. Ecol. 5303–5317 (2021) doi:10.1111/mec.15845. PMC9019111 33590926

[38] Mitchell S. N. Metabolic and target-site mechanisms combine to confer strong DDT resistance in Anopheles gambiae. PLoS One 9, (2014). 10.1371/journal.pone.0092662 PMC3968025 24675797

[39] Riveron J. M. A single mutation in the GSTe2 gene allows tracking of metabolically based insecticide resistance in a major malaria vector. Genome Biol. 15, R27 (2014). 10.1186/gb-2014-15-2-r27 PMC4054843 24565444

[40] Lucas E. R. Copy number variants underlie major selective sweeps in insecticide resistance genes in Anopheles arabiensis. PLoS Biol. 22, e3002898. (2024). 10.1371/journal.pbio.3002898 PMC11620391 39636817

[41] Nagi , 2024. Parallel Evolution in Mosquito Vectors-A Duplicated Esterase Locus is Associated With Resistance to Pirimiphos-methyl in Anopheles gambiae. Mol. Biol. Evol. 41, msae140 (2024). 10.1093/molbev/msae140 PMC11267716 38985692

[42] Laurent B. S. Mosquito vector diversity and malaria transmission. Front. Malar. 1–7 (2025) doi:10.3389/fmala.2025.1600850.

[43] Nagi S. C. & Ingham V. A. A multi-omic meta-analysis reveals novel mechanisms of insecticide resistance in malaria vectors. Commun. Biol. 8, 790 (2025). 10.1038/s42003-025-08221-6 PMC12102355 40410509

[44] Lucas E. R. Whole-genome sequencing reveals high complexity of copy number variation at insecticide resistance loci in malaria mosquitoes. Genome Res. 29, 1250–1261 (2019). 10.1101/gr.245795.118 PMC6673711 31345938

[45] Stevenson B. J. Cytochrome P450 6M2 from the malaria vector Anopheles gambiae metabolizes pyrethroids : Sequential metabolism of deltamethrin revealed. Insect Biochem. Mol. Biol. 41, 492–502 (2011). 10.1016/j.ibmb.2011.02.003 21324359

[46] Lucas E. R. Genome-wide association studies reveal novel loci associated with pyrethroid and organophosphate resistance in Anopheles gambiae and Anopheles coluzzii. Nat. Commun. 14, 4946 (2023). 10.1038/s41467-023-40693-0 PMC10432508 37587104

[47] Kientega M. Genomic insights into the spread and evolution of insecticide resistance variants in Anopheles gambiae s. l. from Burkina Faso. Sci. Rep. 16, 12459 (2026). 10.1038/s41598-026-45950-y PMC13083832 41922661

[48] Li H. & Durbin R. Fast and accurate short read alignment with Burrows-Wheeler transform. Bioinformatics 25, 1754–1760 (2009). 10.1093/bioinformatics/btp324 PMC2705234 19451168

[49] Li H. Aligning sequence reads, clone sequences and assembly contigs with BWA-MEM. Oxford Univ. Press 00, 1–3 (2013).

[50] Musich R., Cadle-Davidson L. & Osier M. V. Comparison of Short-Read Sequence Aligners Indicates Strengths and Weaknesses for Biologists to Consider. Front. Plant Sci. 12, 657240. (2021). 10.3389/fpls.2021.657240 PMC8087178 33936141

[51] Omar R. Z. Digital Commons @ Georgia Southern Mapping next generation sequence data with BWA (Burrows-Wheel Aligner on Galaxy software Mapping next generation sequence data with BWA (Burrows-Wheel Aligner) on Galaxy software. in Mapping next generation sequence data with BWA (Burrows-Wheel Aligner) on Galaxy software 846 (2023).

[52] Mckenna A. The Genome Analysis Toolkit : A MapReduce framework for analyzing next-generation DNA sequencing data. 1297–1303 (2010) doi:10.1101/gr.107524.110.20. PMC2928508 20644199

[53] Martin M. WhatsHap : fast and accurate read-based phasing. bioRxiv Prepr. 1–16 (2016) doi:doi: 10.1101/085050.

[54] Delaneau O., Zagury J., Robinson M. R., Marchini J. L. & Dermitzakis E. T. Accurate, scalable and integrative haplotype estimation. Nat. Commun. 24–29 (2019) doi:10.1038/s41467-019-13225-y. PMC6882857 31780650

[55] MalariaGEN. Selective Whole Genome Amplification (sWGA): Protocol for amplification of Plasmodium falciparum. MalariaGEN 1–6 (2016).

[56] Hudson R. R., Slatkint M. & Wayne P. Estimation of Levels of Gene Flow From DNA Sequence Data. Genet. Soc. Am. 589, 583–589 (1992). 10.1093/genetics/132.2.583 PMC1205159 1427045

[57] Campos M., Rona L. D. P., Willis K., Christophides G. K. & MacCallum R. M. Unravelling population structure heterogeneity within the genome of the malaria vector Anopheles gambiae. BMC Genomics 22, 422 (2021). 10.1186/s12864-021-07722-y PMC8185951 34103015

[58] Mwinyi S. H. Genomic Analysis Reveals a New Cryptic Taxon Within the Anopheles gambiae Complex With a Distinct Insecticide Resistance Profile in the Coast of East Africa. Mol. Ecol. 34, (2025). 10.1111/mec.17762 PMC12051790 40241387

[59] Ranson H. Identification of a point mutation in the voltage-gated sodium channel gene of Kenyan Anopheles gambiae associated with resistance to DDT and pyrethroids. Insect Mol. Biol. 9, 491–497 (2000). 10.1046/j.1365-2583.2000.00209.x 11029667

[60] Mawejje , 2013. Insecticide resistance monitoring of field-collected Anopheles gambiae s.l. populations from Jinja, eastern Uganda, identifies high levels of pyrethroid resistance. Med. Vet. Entomol. 44, 276–283 (2013). 10.1111/j.1365-2915.2012.01055.x PMC3543752 23046446

[61] Ochomo E. Presence of the knockdown resistance mutation, Vgsc-1014F in Anopheles gambiae and An. arabiensis in western Kenya. Parasit. Vectors 8, 615 (2015). 10.1186/s13071-015-1223-5 PMC4666190 26626424

[62] Lehmann T. L. Population Structure of Anopheles gambiae in Africa. Am. Genet. Assoc. 94, 133–147 (2003). 10.1093/jhered/esg024 12721225

[63] Schmidt H. Transcontinental dispersal of Anopheles gambiae occurred from West African origin via serial. Commun. Biol. 2, 473 (2019). 10.1038/s42003-019-0717-7 PMC6923408 31886413

[64] Dennis T. P. W. Cryptic population structure and insecticide resistance in Anopheles gambiae from the southern Democratic Republic of Congo. Sci. Rep. 14, 1–12 (2024). 10.1038/s41598-024-70885-7 PMC11410927 39294180

[65] Ngbolua , 2024. Resistance of Anopheles mosquitoes to insecticides in the Democratic Republic of the Congo : A literature review and future directions. J. Oral Public Heal. 5, e1161 (2024).

[66] Midega J. T. Acta Tropica Population structure of Anopheles gambiae along the Kenyan coast. Acta Trop. 114, 103–108 (2010). 10.1016/j.actatropica.2010.01.011 PMC3775345 20122886

[67] Tangena Indoor residual spraying for malaria control in sub - Saharan Africa 1997 to 2017 : an adjusted retrospective analysis. Malar. J. 19, 150 (2020). 10.1186/s12936-020-03216-6 PMC7149868 32276585

[68] Diarra M. High resolution physically based modelling reveals malaria incidence reduction by vector control measures. Sci. Rep. 16, 1288 (2026). 10.1038/s41598-025-33539-w PMC12791136 41507284

[69] Sherrard-smith E. Articles Optimising the deployment of vector control tools against malaria : a data-informed modelling study. Lancet 6, e100–09 (2018). 10.1016/S2542-5196(21)00296-5 35065707

[70] Rosser N., Seixas F. & Mallet J. Sympatric speciation by allochrony ? Mol. Ecol. 31, 3975–3978 (2022). 10.1111/mec.16599 35789010

[71] Kamau A. Variation in the effectiveness of insecticide treated nets against malaria and outdoor biting by vectors in Kilifi, Kenya [version 4 ; referees : 1 approved, 3 approved with reservations] Referee Status : Wei Sheng Yan Jiu 2, 22 (2018). 10.12688/wellcomeopenres.11073.1 PMC6281023 30542660

[72] Edi C. V. CYP6 P450 Enzymes and ACE-1 Duplication Produce Extreme and Multiple Insecticide Resistance in the Malaria Mosquito Anopheles gambiae. PLoS Genet. 10, e1004236 (2014). 10.1371/journal.pgen.1004236 PMC3961184 24651294

[73] Hancock P. A., Ochomo E. & Messenger L. A. Genetic surveillance of insecticide resistance in African Anopheles populations to inform malaria vector control. Trends Parasitol. 40, 604–618 (2024). 10.1016/j.pt.2024.04.016 38760258

[74] Etang J. Patterns of Kdr-L995F Allele Emergence Alongside Detoxifying Enzymes Associated with Deltamethrin Resistance in Anopheles gambiae s.l. from North Cameroon. Pathogens 11, 253 (2022). 10.3390/pathogens11020253 PMC8876678 35215196

