# Supplementary material for: Genomic Connectivity and Adaptive Gene Flow of Anopheles gambiae s. s. Across East and Central Africa
Source: Res Sq. 2026 Oct 1:rs.3.rs-10571451. Preprint. [Version 1] doi: 10.21203/rs.3.rs-10571451/v1 (PMC13648852; doi:10.21203/rs.3.rs-10571451/v1)
Supplement: Supplementary file 1 [file NIHPPrs10571451V1-supplement-1.pdf]

Supplementary Figures

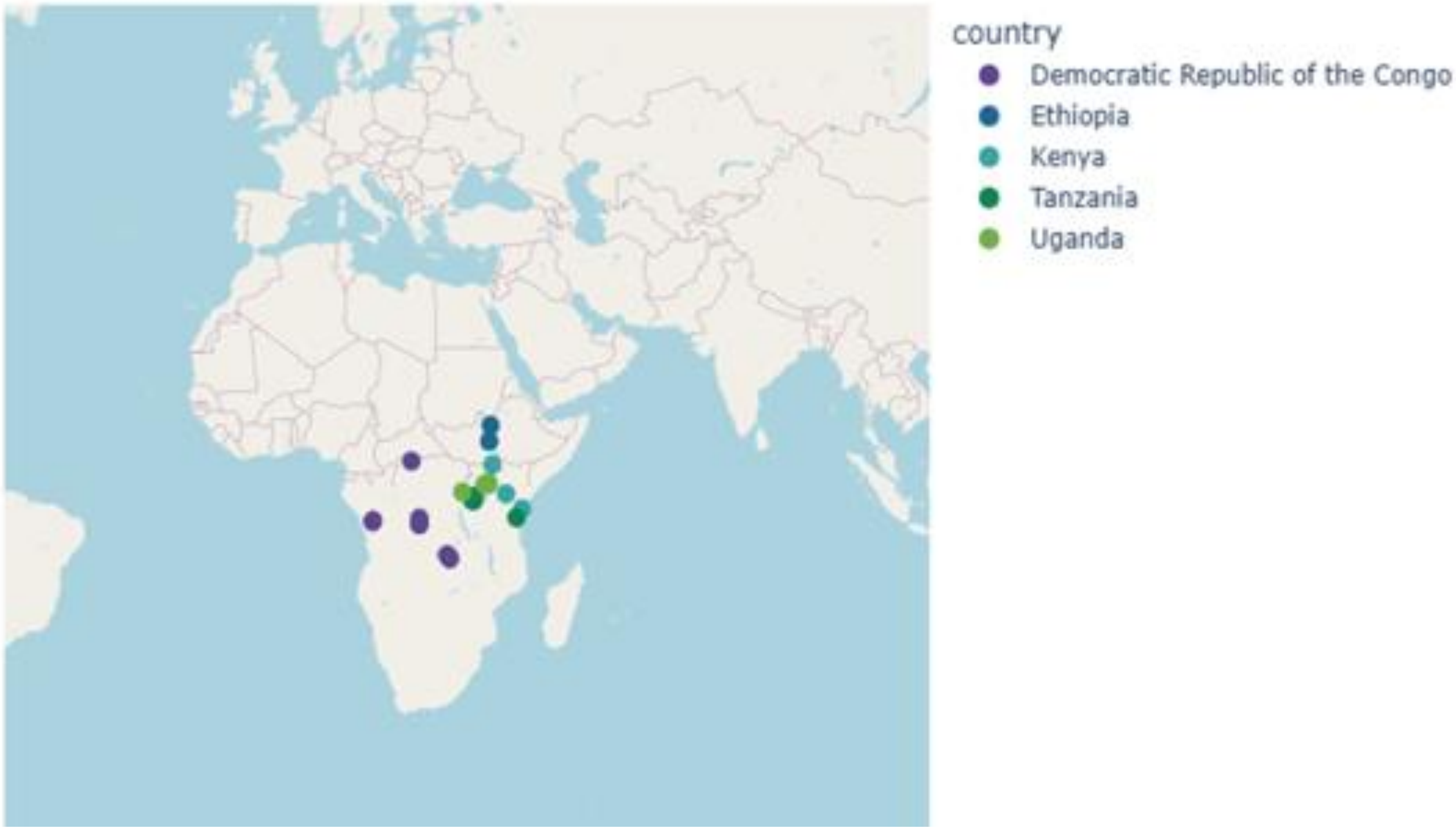

Figure S1. Map of the study regions showing ecological zones.

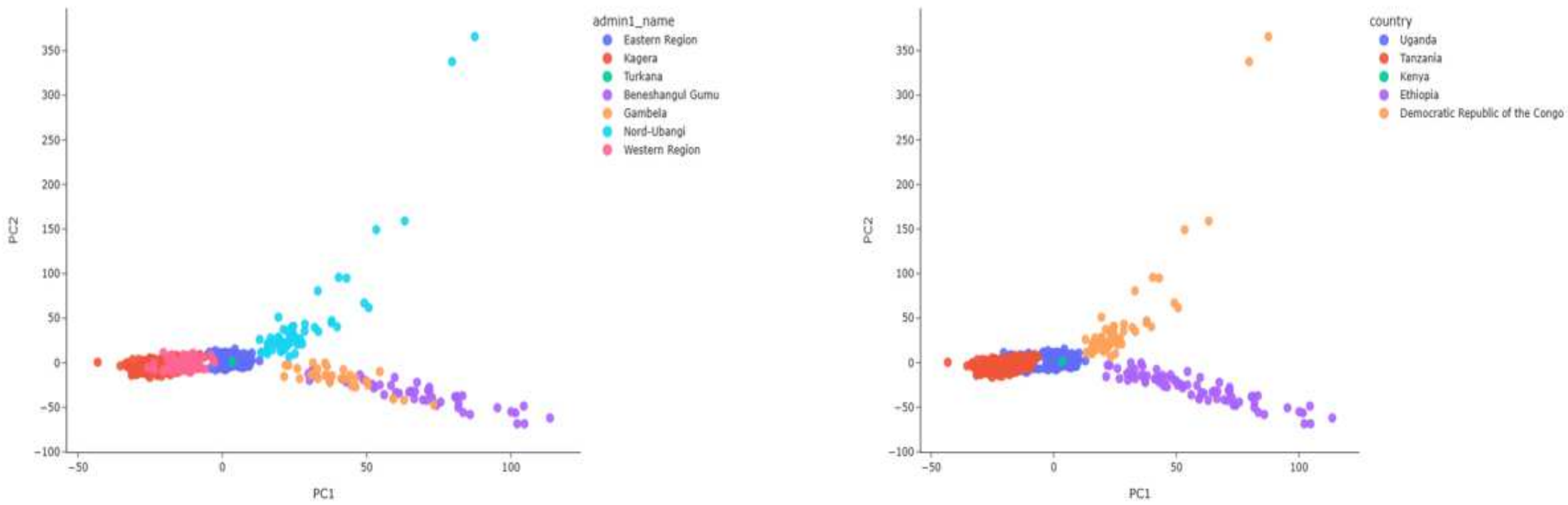

Figure S2. Further analysis of the biggest cluster in principal component analysis (PCA) plot of the inland regions across East and Central African *Anopheles gambiae* populations for the fine-scale differentiation.

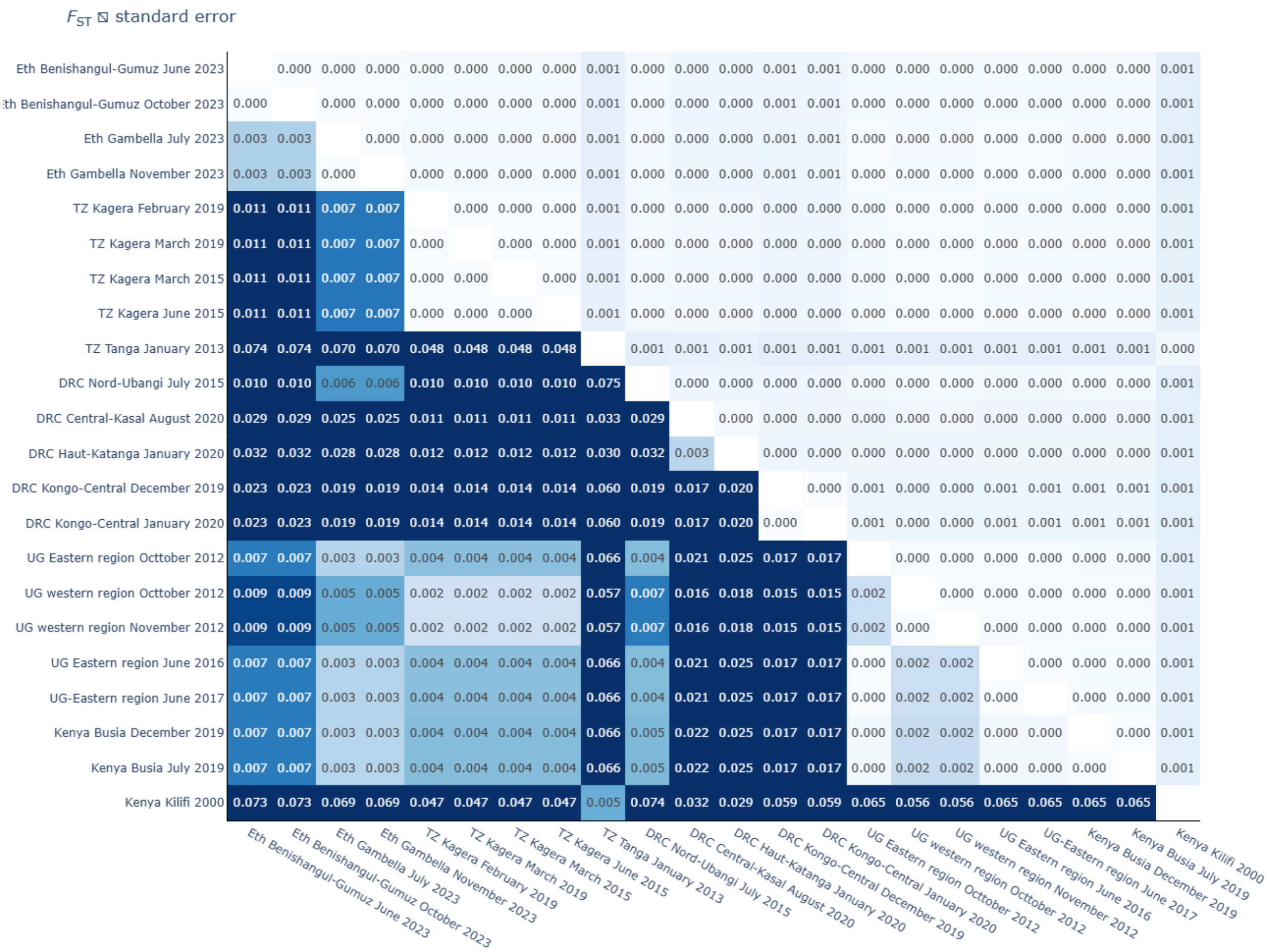

Figure S3. ***Pairwise genetic differentiation (F_{ST})*** **among** ***Anopheles gambiae*** **populations across East and Central African regions, with darker shades of blue indicating higher values.**

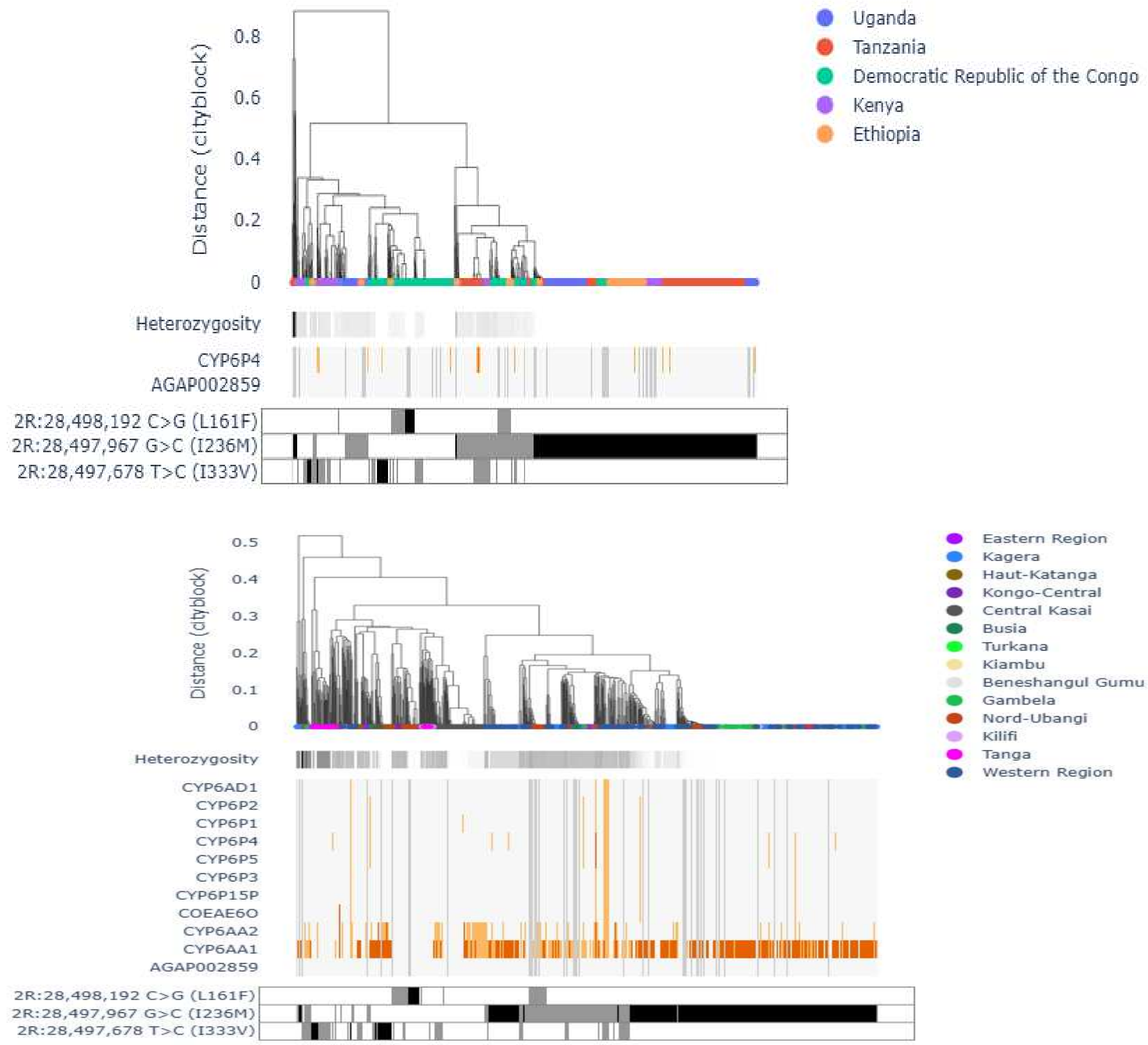

Figure S4. Diplotype clustering of *Anopheles gambiae* samples across East and Central African *Anopheles gambiae* populations within the *Cyp6aa/p* gene cluster on chromosome 2R.

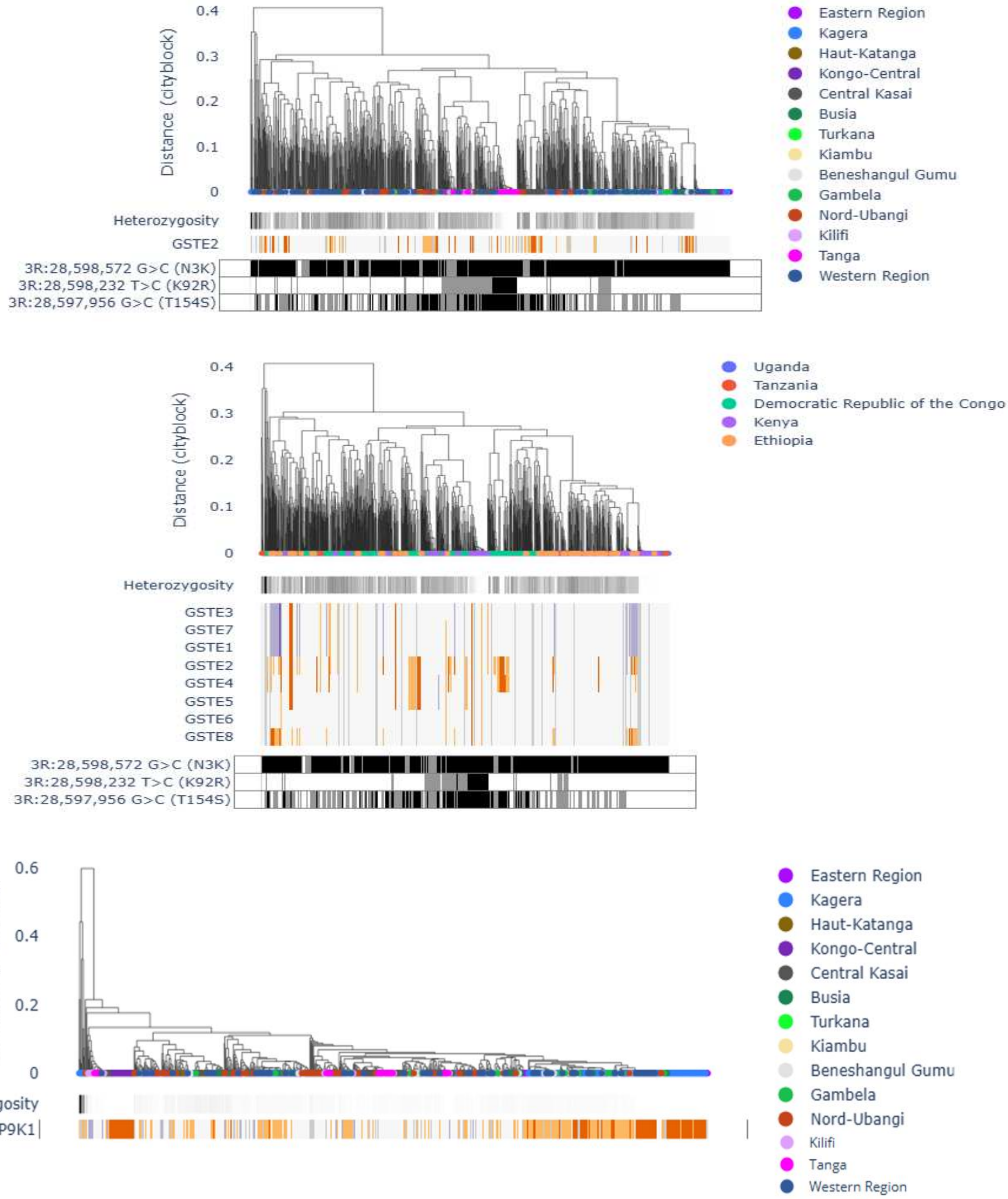

Figure S5. Diplotype clustering of *Anopheles gambiae* samples across East and Central African *Anopheles gambiae* populations within the *Cyp6aa/p* gene cluster on chromosome 3R contigs.

Supplementary Tables

Table S1. Mosquito samples used in analysis

Country	Locations	Year	Month	Sample number	Total
Uganda	Eastern	2026	June	24	252
		2027	June	21	
		2012	October	112	
	Western	2012	October	49	
			November	46	
Tanzania	Kagera	2015	March	13	291
			June	19	
		2019	February	12	
			March	215	
	Tanga	2013	January	32	
Kenya	Kilifi	2000	January	19	50
	Busia	2019	July	14	
	Turkana	2006	February	1	
	Kiambu	2019	July	1	
Ethiopia	Gambella	2023	July	7	31
			November	1	
	Benishangul-Gumuz		June	12	48
			October	36	
DRC	Central Kasai	2020	August	77	241
	Haut-Katanga		January	46	
	Congo-central	2019	December	20	
		2020	January	22	
	Nord-Ubangi	2015	July	76	
Total					913

Table S2. The sample sets and accession numbers for *Anopheles gambiae* sequence data.

**Sample sets**	**Accession numbers**
1324-VO-ET-GOLASSA-VMF00257	ERR12314211-ERR12314243
1324-VO-ET-GOLASSA-VMF00257	ERR12326237-ERR12326277
1324-VO-ET-GOLASSA-VMF00275	ERR12983154-ERR12983193
1324-VO-ET-GOLASSA-VMF00275	ERR13101381-ERR13101432
